# C-Reactive Protein-to-Albumin Ratio (CAR) and Left Atrial Diameter Predicts New-Onset Atrial Fibrillation in Chronic Coronary Syndrome: A Retrospective Cohort Study

**DOI:** 10.3390/jcm15010255

**Published:** 2025-12-29

**Authors:** Xiaoying Xie, Jingjing Chen, Liangying Lin, Ximei Zhang, Baoshun Hao, Shujie Yu, Yesheng Ling, Xiaoxian Qian, Shaojie Lai, Yong Liu, Lin Wu, Bin Zhou

**Affiliations:** 1 Department of Cardiology, The Third Affiliated Hospital, Sun Yat-Sen University, Guangzhou 510000, China; xiexy227@mail2.sysu.edu.cn (X.X.); zhandxm226@mail.sysu.edu.cn (X.Z.); haobsh@mail.sysu.edu.cn (B.H.); yushujie@mail.sysu.edu.cn (S.Y.); lingysh5@mail.sysu.edu.cn (Y.L.); qianxx@mail.sysu.edu.cn (X.Q.); laishj6@mail2.sysu.edu.cn (S.L.); liuy693@mail.sysu.edu.cn (Y.L.); 2 Big Data and Artificial Intelligence Center, The Third Affiliated Hospital of Sun Yat-Sen University, Guangzhou 510000, China; chenjj336@mail.sysu.edu.cn; 3 Department of Hospital Infection Control, The Third Affiliated Hospital of Sun Yat-Sen University, Guangzhou 510000, China; linly35@mail.sysu.edu.cn

**Keywords:** new-onset atrial fibrillation, chronic coronary syndrome, left atrial diameter, C-reactive protein to albumin ratio, predictive model

## Abstract

**Background/Objectives:** New-onset atrial fibrillation (NOAF) frequently develops in patients with chronic coronary syndrome (CCS) and is associated with adverse cardiovascular outcomes. The C-reactive protein–to–albumin ratio (CAR) reflects systemic inflammation, whereas left atrial diameter (LAD) indicates structural cardiac remodeling. Their combined predictive role for NOAF in CCS remains uncertain. This study evaluated the predictive value of combined CAR and LAD for NOAF in CCS patients. **Methods**: We retrospectively analyzed 2431 CCS patients treated at the Third Affiliated Hospital of Sun Yat-sen University between 2012 and 2019. The primary endpoint was NOAF occurrence during follow-up. Receiver operating characteristic (ROC) analysis determined exploratory cutoff values for CAR (0.0429) and LAD (33.96 mm). Patients were categorized into four groups: Group 1 (low CAR–low LAD), Group 2 (high CAR–low LAD), Group 3 (low CAR–high LAD), and Group 4 (high CAR–high LAD). Cox proportional hazards, Kaplan-Meier, and subgroup analyses were conducted to evaluate associations with NOAF risk. **Results:** During a median follow-up of 4.96 years, 93 NOAF events were identified. Compared with the Group 1, patients with higher CAR and LAD showed significantly elevated NOAF risk (HR = 2.67, 95%CI 1.99–3.57, *p* < 0.001). The combined CAR–LAD model demonstrated superior predictive accuracy (AUC = 0.731, 95% CI = 0.654–0.765; *p* < 0.001) and consistent effects across most subgroups. Decision curve analysis confirmed greater net clinical benefit for the combined model. **Conclusions**: The integration of CAR and LAD serves as a simple, non-invasive, and effective tool for predicting NOAF in CCS patients. This dual-marker model facilitates early identification of high-risk individuals and support personalized preventive strategies in clinical practice.

## 1. Introduction

Chronic coronary syndrome (CCS)—the long-term, dynamic clinical manifestation of coronary artery disease (CAD)—remains a major global burden [1]. Globally, CAD affected approximately 254 million people and caused about 9 million deaths in 2021, underscoring the need for robust risk stratification in chronic CAD populations [2]. Within CCS, new-onset atrial fibrillation (NOAF), defined as first-detected atrial fibrillation (AF) or atrial flutter (AFL) in individuals without prior AF/AFL history, represents a common and clinically significant complication that confers excess risks of stroke, heart failure, and overall mortality [3]. Among ambulatory CCS populations, NOAF develops at a rate of roughly 1.12 per 100 person-years in this population (95% CI 1.06–1.18), with a 5-year cumulative incidence of about 5%; estimates vary by setting, and outpatient stable CAD cohorts have reported rates around 1.6% per year [4,5]. In China, nationwide CCS-specific NOAF incidence has not yet been reported; however, an adult AF prevalence of roughly 1.6% indicates a substantial at-risk CCS population and emphasizes the need for early risk identification, targeted prevention, and structured follow-up [6]. Existing research on NOAF within CAD has largely emphasized acute and interventional settings, including acute coronary syndrome, heart failure, percutaneous coronary intervention, and coronary artery bypass grafting, where its occurrence is a well-established predictor of adverse outcomes, highlighting an evidence gap in chronic, ambulatory CCS [6,7,8,9]. These gaps motivate the search for simple, scalable biomarkers to stratify. NOAF risk specifically in CCS and to guide surveillance strategies. Although CCS represents a chronic manifestation of CAD, its distinct pathophysiological features—characterized by persistent ischemia, long-term hemodynamic burden, and low-grade inflammation—may lead to different mechanisms and predictors of NOAF compared with acute CAD settings.

The mechanisms of NOAF in CAD conform to a classic “trigger–substrate” paradigm: ischemia, oxidative stress, inflammation, and sympathetic activation serve as triggers, whereas chronic pressure or volume overload, atrial fibrosis, and electrical–structural remodeling constitute the substrate [10,11,12]. Most evidence arises from ACS cohorts, in which acute inflammation amplifies NOAF risk [13]; however, large CCS registries such as CLARIFY have confirmed that NOAF also occurs in CCS outpatients and is associated with adverse cardiovascular prognosis [4]. By contrast, CCS is characterized by chronic low-grade inflammation and progressive remodeling [1], limiting direct extrapolation from ACS uncertainty and underscoring the need for CCS-specific prediction studies.

In CCS, persistent low-grade inflammation promotes ectopic activity and conduction heterogeneity, facilitating AF initiation on a vulnerable atrial substrate. Notably, large stable coronary disease cohorts have demonstrated that residual inflammatory risk, reflected by CRP-based markers, persists despite optimal secondary prevention and remains strongly associated with adverse cardiovascular outcomes [14,15]. The C-reactive protein-to-albumin ratio (CAR), derived from the combination of C-reactive protein (CRP, a positive acute-phase reactant) and albumin (a negative acute-phase protein), represents a composite marker of systemic inflammation and nutritional status, and has demonstrated greater prognostic relevance than either biomarker alone [16]. Elevated CAR independently predicts perioperative or in-hospital NOAF and adverse outcomes in cardiac surgery, supporting its relevance to arrhythmic risk [17]. Chronic hemodynamic stress leads to left atrial dilation, fibrosis, and conduction slowing, which are hallmarks of an arrhythmogenic substrate [18]. Left atrial diameter (LAD), a widely available echocardiographic parameter of atrial volume load and structural remodeling, has emerged as an independent determinant of incident AF and adverse cardiovascular prognosis [16,17]. Consistent with progressive atrial remodeling in CCS, recent studies show that left atrial functional impairment detected by speckle-tracking strain can be present in some CCS patients before overt chamber enlargement, supporting the presence of an evolving atrial substrate in chronic CAD [19]. CAR and LAD, respectively, represent the inflammatory “trigger” and the anatomical “substrate,” providing complementary, low-cost, and clinically feasible indicators for risk stratification.

Therefore, this study investigated predictive role of CAR, LAD, and their combination to predict NOAF in CCS patients. We further contrasted their predictive capacity with conventional risk factors and to explore whether the CAR–LAD combination could provide incremental prognostic utility in this population.

## 2. Methods

### 2.1. Study Design and Population

**Figure 1:**
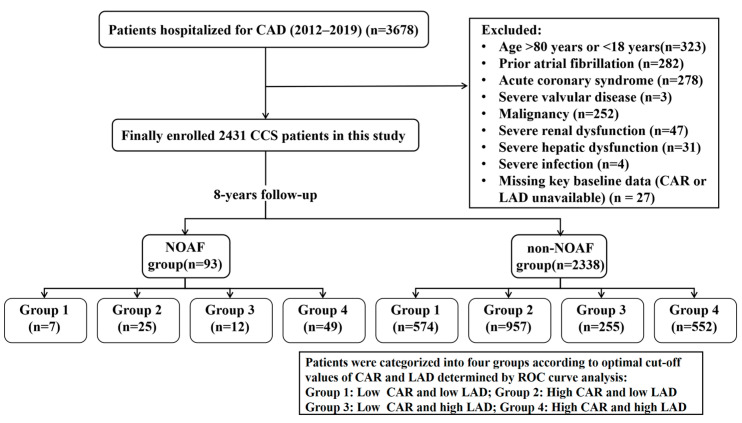
Flowchart of the study population. CAD, Coronary artery disease; CCS, Chronic coronary syndrome. NOAF, new-onset atrial fibrillation. LAD, Left atrial diameter. CAR, C-reactive protein to albumin ratio.

The present study is a single-center retrospective observational study that was conducted at the Third Affiliated Hospital of Sun Yat-sen University from January 2012 to December 2019. Patients between 18 and 80 years of age, diagnosed with suspected or established CCS according to the 2019 European Society of Cardiology (ESC) guidelines [20], were considered eligible. In our centre, patients with CCS are typically hospitalised for comprehensive diagnostic evaluation, optimisation of pharmacological therapy, or preparation for potential revascularisation procedures. Hospitalisation is also indicated when symptoms worsen, when non-invasive tests suggest a high-risk profile (e.g., markedly positive stress test results or reduced left ventricular function), or when closer monitoring is required to ensure patient safety. Patients with CAD were eligible if they fulfilled at least one of the following criteria: (1) prior myocardial infarction occurring >3 months before enrollment; (2) angina with evidence of myocardial ischemia; (3) coronary angiography demonstrating ≥1 stenosis >50%; (4) history of CABG or PCI performed >3 months prior to enrollment. The main exclusion criteria included: (1) history of AF or AFL; (2) thyroid dysfunction; (3) malignant tumour; (4) severe infection or hepatic/renal insufficiency; (5) incomplete clinical data and follow-up; (6) other severe cardiovascular diseases, such as advanced heart failure, significant valvular disorders, or prior valve surgery/replacement. The detailed patient selection process, including the number of patients screened, excluded at each stage with specific reasons, and included in the final analysis, is shown in Figure 1. Ethical approval was obtained from the Third Affiliated Hospital of Sun Yat-sen University (No. (2025)-382-02), and all procedures adhered to the Declaration of Helsinki.

### 2.2. Data Sources and Operational Definitions

Baseline demographic, clinical, echocardiographic, and laboratory variables were retrospectively obtained from hospital electronic records. Data encompassed age, sex, vital parameters, relevant medical history, coronary angiographic findings, echocardiographic measurements, and standard laboratory tests (e.g., lipid profile, glucose metabolism, renal and liver function, inflammatory markers, and hematological parameters). All laboratory measurements were obtained from venous blood samples collected in the morning of the day of index hospitalization after an overnight fast. Remnant lipoprotein cholesterol (RLPC) was calculated as TC−LDL-C−HDL-C, and the CAR as CRP (mg/L) ÷ albumin (g/L). LAD was obtained via transthoracic echocardiography in the parasternal long-axis plane. Receiver operating characteristic (ROC) curve analysis was applied to define the exploratory cut-offs for CAR and LAD, and patients were subsequently classified into high- and low-level groups. Detailed definitions of study variables are provided in Table S2.

### 2.3. Outcomes

For this study, NOAF referred to the initial documented occurrence of AF or AFL in individuals without a prior history, identified during follow-up. Diagnosis of NOAF was confirmed through (1) a 12-lead electrocardiogram (ECG) obtained at routine annual or unscheduled follow-up visit, or (2) ICD-10 coded discharge diagnoses for AF (I48.0, I48.1, I48.2, I48.9) or AFL (I48.3, I48.4) at the index hospitalization, with no prior AF/AFL codes recorded within the last 8 years. ICD-10 codes included I48.0, I48.1, I48.2, I48.3, I48.4, and I48.9; corresponding ICD-9 codes were also screened during the earlier study period. During each visit, a trained physician assessed cardiac rhythm, with the timing of NOAF diagnosis recorded accordingly. To ensure diagnostic validity, all NOAF cases identified through administrative codes were systematically verified by reviewing inpatient 12-lead ECGs, Holter, or continuous cardiac monitoring data when available, and physician progress notes documenting AF or AFL episodes in the electronic medical records. CCS patients who experienced ≥1 confirmed AF or AFL event during follow-up (NOAF group) were contrasted with those remaining in sinus rhythm over 8-year follow-up period. This multi-source verification strategy has been routinely used in our institution and is expected to yield a high positive predictive value for NOAF ascertainment. Details of ICD code validation are provided in Method S1.

### 2.4. Follow-Up

All patients were followed from the date of index hospitalisation until the occurrence of NOAF or the end of the study period, whichever came first.

### 2.5. Statistical Analysis

Normally distributed continuous variables were summarized as mean ± standard deviation (SD) and compared with the Student’s *t*-test. Skewed variables were reported as median with interquartile range (IQR) and analyzed using the Mann–Whitney U test. Categorical variables were described as frequencies (percentages) and compared using either the χ^2^ test or Fisher’s exact test, as appropriate. Given the low proportion of missing data (<5%), missing values were handled using single imputation prior to further analyses, with continuous variables imputed by the mean and categorical variables imputed by the mode. ROC analysis was performed to determine exploratory CAR and LAD thresholds for NOAF prediction, with the Youden index applied to maximize sensitivity and specificity. According to these thresholds, patients were subsequently classified into four groups: Group 1 (low CAR–low LAD), Group 2 (high CAR–low LAD), Group 3 (low CAR–high LAD), and Group 4 (high CAR–high LAD). Cox proportional hazards models were applied to assess the relationships of LAD, CAR, and their combination with the risk of NOAF. Hazard ratios (HRs) with 95% confidence intervals (CIs) were estimated. The proportional hazards assumption for all Cox models was verified using Schoenfeld residual tests, with all covariates satisfying the assumption (all *p* > 0.05). Three regression models were specified: Model 1 was unadjusted; Model 2 included adjustment for age and sex; and Model 3 was additionally adjusted for a range of clinically relevant covariates. including systolic blood pressure (SBP), apolipoprotein B100 (ApoB100), creatinine, serum potassium (K^+^), uric acid (UA), total serum cholesterol (TC), glycated hemoglobin (HbA1c), pulse rate, left ventricular ejection fraction (LVEF), and histories of alcohol consumption, diabetes mellitus, stroke, and hyperhomocysteinemia. Proportional hazards were assessed in all models. Cumulative NOAF incidence by LAD–CAR category was analyzed using Kaplan–Meier curves, with group differences assessed via the log-rank test. Subgroup analyses were conducted to assess whether associations were consistent across clinically relevant strata. Decision curve analysis (DCA) was conducted to assess the net clinical benefit of predictive models incorporating CAR and LAD. Statistical analyses were carried out using SPSS 26.0 (IBM Corp., Armonk, NY, USA) and R 4.3.1 (R Foundation for Statistical Computing, Vienna, Austria). Statistical significance was defined as a two-sided *p* < 0.05. The analysis code used in this study is available from the corresponding author upon reasonable request. Details of missing data patterns and the imputation strategy are provided in Method S2.

## 3. Result

### 3.1. Baseline Demographic, Clinical, and Laboratory Features

The study sample flow diagram is presented in Figure 1. During a median follow-up of 4.96 years (IQR 3.15–6.33), 93 of the 2431 CCS patients, including 58.1% males, with a mean age 67 years. Detailed baseline features are presented in Table 1. Compared with patients without NOAF, those who developed NOAF were significantly older (74.0 [69.0;77.0] vs. 67.0 [60.0;74.0] years, *p* < 0.001) and had lower diastolic blood pressure (DBP: 74.0 [70.0;82.0] vs. 78.0 [70.0;86.0] mmHg, *p* = 0.031). Regarding lipid metabolism, NOAF patients had significantly lower TC (4.09 [3.38;4.72] vs. 4.38 [3.60;5.29] mmol/L, *p* = 0.003), triglycerides (TG: 1.16 [0.89;1.53] vs. 1.32 [0.93;1.87] mmol/L, *p* = 0.012), low-density lipoprotein cholesterol (LDL-C: 2.53 [1.82;3.00] vs. 2.68 [2.02;3.41] mmol/L, *p* = 0.022), reversible lipid-protein complexes (RLPC: 0.45 [0.32;0.61] vs. 0.52 [0.34;0.78] mmol/L, *p* = 0.025), and ApoB100 (0.95 [0.69;1.17] vs. 1.02 [0.79;1.30] mmol/L, *p* = 0.003) compared with non-NOAF patients, indicating an overall hypolipidemic profile. Additionally, markers of renal function and systemic inflammation were higher in the NOAF group, including blood urea nitrogen (BUN: 6.50 [5.14;8.16] vs. 5.58 [4.57;6.99] mmol/L, *p* < 0.001), serum creatinine (Cr: 85.0 [72.0;106.0] vs. 79.0 [65.9;95.0] μmol/L, *p* = 0.003), C-reactive protein (CRP: 10.4 [2.09;32.5] vs. 4.20 [1.00;22.3] mg/L, *p* = 0.003), and the CRP-to-albumin ratio (CAR: 0.24 [0.05;0.91] vs. 0.11 [0.02;0.59], *p* = 0.004). Echocardiographic assessment revealed larger left atrial diameter (LAD) in NOAF patients (36.0 [33.0, 40.0] vs. 32.0 [30.0, 36.0] mm, *p* < 0.001).

### 3.2. ROC Analysis and Baseline Characteristics by CAR–LAD Groups

**Figure 2:**
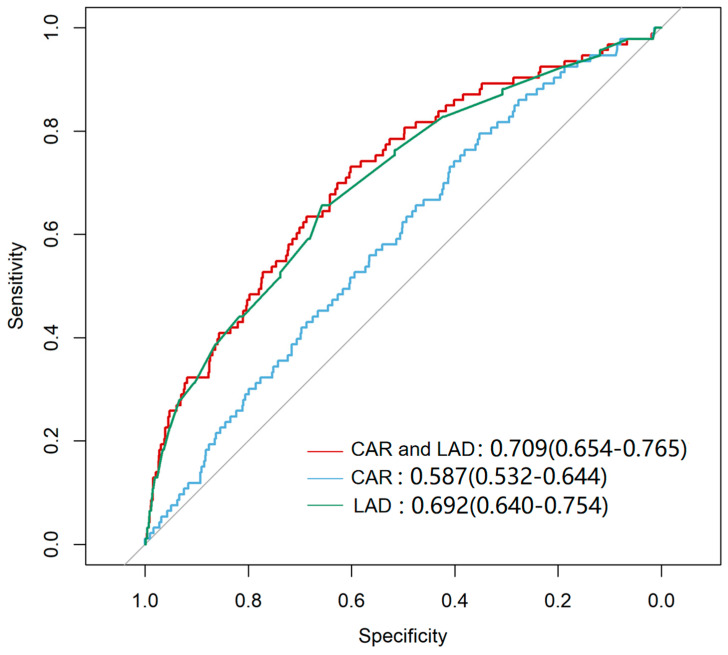
Receiver Operating Characteristic (ROC) curve for Predicting NOAF. The curves represent the predictive performance of LAD alone, CAR alone, and the combination of LAD and CAR. The combined model LAD and CAR demonstrated the highest sensitivity and specificity for predicting NOAF (*p* < 0.001). The diagonal line represents the reference line indicating no discrimination (AUC = 0.5).

ROC curve analysis evaluated the predictive ability of CAR, LAD, and their combination for NOAF (Figure 2). ROC-derived cut-offs were CAR ≥ 0.0429 and LAD ≥ 33.96 mm (Table 2). The combination of CAR and LAD yielded the highest predictive accuracy (AUC = 0.709, 95% CI: 0.654–0.765; *p* < 0.001), followed by LAD alone (AUC = 0.692, 95% CI: 0.640–0.754; *p* < 0.001) and CAR alone (AUC = 0.587, 95% CI: 0.532–0.644; *p* = 0.002). Based on exploratory thresholds, patients were grouped into four groups (Groups 1–4). Compared with Group 1, the other subgroups were generally older, exhibited higher SBP, showed greater prevalence of hypertension and diabetes, and had lower rates of hyperlipidemia. Notably, participants in Group 4 showed higher levels of blood urea nitrogen (BUN), Cr, UA, HbA1c, and the triglyceride-glucose (TyG) index, while significant differences in TC, HDL-C, LDL-C, Lp(a), and ApoB100 were observed across the four groups (Table 3).

### 3.3. Cox Proportional Hazards Analysis of CAR and LAD for Predicting NOAF

Cox regression analyses (Table 4) revealed that higher CAR was linked to greater NOAF risk. In the unadjusted model, a one-unit rise in CAR corresponded to a 2.02-fold higher hazard of NOAF (HR = 2.02, 95% CI: 1.22–3.36, *p* = 0.006). This association persisted after adjustment for age and sex (Model 2) and further adjustment for multiple covariates (Model 3), with a fully adjusted HR of **1.85** (95% CI, 1.11–3.08; *p* = 0.018). An enlarged LAD predicted greater NOAF risk, with each additional 1 mm linked to a 13% higher hazard in the unadjusted model (HR = 1.13, 95% CI: 1.10–1.17, *p* < 0.001). Furthermore, LAD ≥33.96 mm conferred a 3.39-fold increased risk compared to smaller LAD (HR = 3.39, 95% CI: 2.21–5.20, *p* < 0.001). This association remained significant in both age- and sex-adjusted (Model 2) and multivariable-adjusted (Model 3) analyses, with a fully adjusted HR of 2.87 (95% CI, 1.84–4.48; *p* < 0.001).

Combining CAR and LAD revealed a graded increase in NOAF risk (Table 4), with the high CAR-high LAD group (Group 4) exhibiting the highest risk of NOAF compared with the low CAR–low LAD group (Group 1). In the unadjusted model, patients in Group 4 showed a 6.52-fold higher risk of NOAF (HR = 6.52, 95% CI: 2.95–14.39, *p* < 0.001). This association persisted after adjustment for age and sex and other covariates (fully adjusted HR = 5.05, 95% CI: 2.26–11.31, *p* < 0.001), with a significant positive trend across categories (*p* for trend < 0.001).

### 3.4. Kaplan–Meier Survival Analysis of NOAF-Free Survival by CAR, LAD, and the CAR–LAD Combination

**Figure 3:**
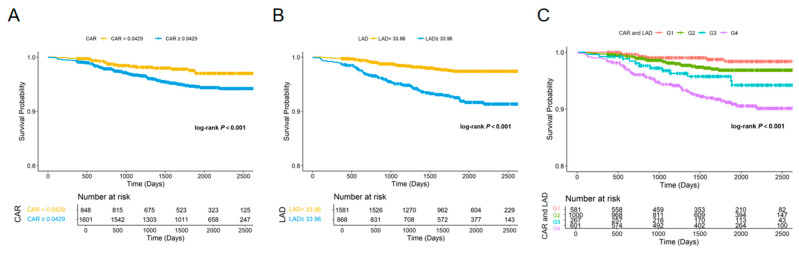
Kaplan–Meier curves for NOAF according to CAR, LAD, and their combination. (**A**) Kaplan–Meier estimates of NOAF-free survival stratified by CAR (<0.0429 vs. ≥0.0429). (**B**) Kaplan–Meier estimates stratified by LAD (<33.96 mm vs. ≥33.96 mm); (**C**) Kaplan–Meier estimates stratified by the combined CAR–LAD categories: Group 1, low CAR–low LAD; Group 2, high CAR–low LAD; Group 3, low CAR–high LAD; and Group 4, high CAR–high LAD. The number of patients at risk is shown below each plot. Log-rank tests were used to compare survival distributions, and all comparisons were statistically significant (*p* < 0.001).

Kaplan–Meier survival analysis was performed to evaluate NOAF-free survival according to CAR, LAD, and their combination. Patients with higher CAR (≥0.0429) exhibited a significantly higher incidence of NOAF compared with those with lower CAR (<0.0429) (log-rank *p* < 0.001; Figure 3A). Similarly, a larger LAD (≥33.96 mm) was associated with a marked reduction in NOAF-free survival relative to a smaller LAD (<33.96 mm) (log-rank *p* < 0.001; Figure 3B).

When CAR and LAD were combined, a clear stratification of NOAF risk was observed among the four groups (log-rank *p* < 0.001; Figure 3C). Individuals in Group 4 (high CAR and high LAD) had the highest incidence of NOAF, followed by Group 3 (low CAR and high LAD) and Group 2 (high CAR and low LAD), whereas Group 1 (low CAR and low LAD) had the best prognosis.

### 3.5. Subgroup Analysis

**Figure 4:**
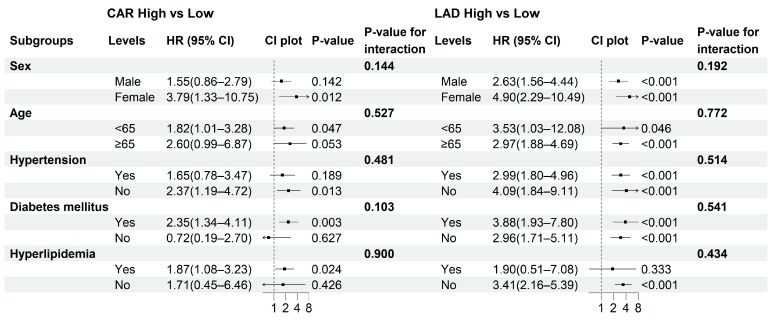
Subgroup analyses of the associations between CAR and LAD with NOAF. Forest plots display the hazard ratios (HRs) and 95% confidence intervals (CIs) for high versus low CAR (**left panel**) and LAD (**right panel**) across predefined subgroups. *p*-values indicate within-stratum effects, and *p* for interaction reflects the significance of the multiplicative interaction between each exposure and the subgroup variable. Arrows indicate that the corresponding hazard ratios or confidence intervals extend beyond the limits of the plotted axis.

Subgroup analyses were performed to evaluate the robustness of the associations of CAR and LAD with NOAF across clinically relevant strata (sex, age, diabetes mellitus, hypertension, and hyperlipidemia). As shown in Figure 4, the associations remained consistent across most subgroups, with no significant effect modification detected (*p* for interaction > 0.05 for all). The risk of NOAF associated with high CAR or high LAD was particularly evident among males, participants aged ≥ 65 years, and non-diabetic or non-hypertensive individuals. In an exploratory analysis using the combined CAR–LAD categories (Group 1–4), results were directionally similar, although some strata yielded wide confidence intervals due to sparse events (Table S1, Supplementary Materials).

### 3.6. Decision Curve Analysis

**Figure 5:**
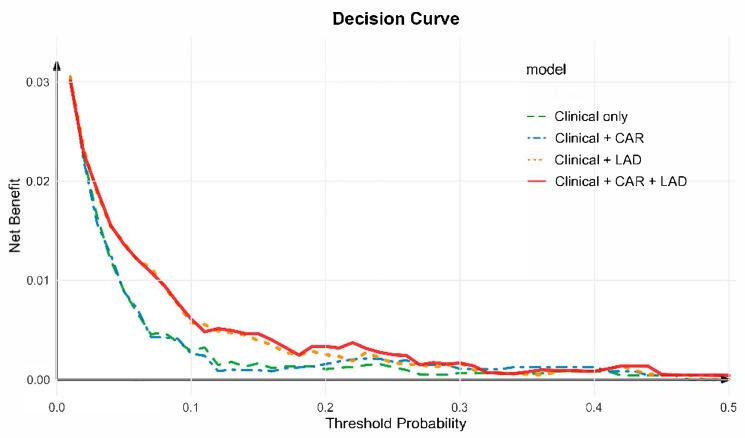
Decision curve analysis for predicting new-onset atrial fibrillation. Decision curve analysis (DCA) was used to evaluate the clinical utility of different predictive models for NOAF. The clinical model (green line) was adjusted for age, sex, systolic blood pressure, apolipoprotein B100, creatinine, potassium, uric acid, total cholesterol, glycated hemoglobin, drinking history, diabetes mellitus history, pulse, left ventricular ejection fraction (LVEF), stroke history, and hyperhomocysteinemia history. The combined model incorporating CAR and LAD (red line) achieved the highest net benefit across most threshold probabilities, indicating improved predictive performance compared with the clinical-only and single-marker models.

Decision curve analysis (DCA) was performed to evaluate the net clinical benefit of adding CAR and LAD to the conventional clinical model. As shown in Figure 5, across a wide range of threshold probabilities, models incorporating CAR + LAD yielded greater net benefit than the clinical model alone. The combined model consistently provided the highest overall net benefit, followed by the Clinical + LAD and Clinical + CAR models, whereas the clinical-only model showed the lowest utility. These findings indicate that incorporating both CAR and LAD can improve individualized risk stratification and clinical decision-making for NOAF prediction.

## 4. Discussion

The present study demonstrated that integrating inflammatory and structural parameters significantly improved the prediction of NOAF in patients with CCS. Specifically, the combined model incorporating CAR and LAD showed superior discriminative performance compared with either marker alone, and the DCA further confirmed its greater net clinical benefit across a broad range of threshold probabilities. These findings suggest that systemic inflammation and atrial remodeling, instead of functioning independently, may interact to promote atrial arrhythmogenesis [19,21,22].

Collectively, these observations extend the conventional concept that atrial fibrillation in CCS is primarily driven by structural or electrical remodeling alone [23]. Rather, they point to a close association between inflammatory activity and atrial enlargement, in which higher inflammatory burden tends to coexist with more atrial structural deterioration and electrical instability [24]. The rationale for combining CAR and LAD lies in their complementary biological significance: CAR quantifies the systemic inflammatory burden as an upstream “trigger,” whereas LAD reflects the structural substrate that sustains arrhythmia. Integrating these two dimensions captures both inflammatory activation and anatomical remodeling, aligning with the dual trigger–substrate hypothesis of AF development. This interplay carries important clinical relevance; however, given the observational nature of this study, these findings should be interpreted as associations rather than evidence that modifying inflammatory stress would attenuate structural deterioration or reduce NOAF risk in CCS.

In this CCS cohort, NOAF occurred in 3.80% of patients, consistent with the 5.0% reported in the CLARIFY registry [4], supporting the external validity of our findings. We identified an exploratory CAR cutoff of 0.0429 for predicting NOAF, lower than the 0.0533 threshold reported in broader cardiovascular populations [25]. This suggests that in CCS, characterized by chronic low-grade inflammation and atherosclerosis, NOAF may develop even under modest systemic inflammatory stress. Clinically, it indicates that mild CAR elevation warrants attention in seemingly stable patients, although this threshold should be interpreted cautiously given the modest discriminatory performance of CAR alone. The lower CAR threshold observed in this study may reflect the distinct inflammatory background of CCS and complements, rather than replaces, structural or electrical remodeling markers. It highlights that inflammation may serve as an early marker of atrial vulnerability rather than a mere bystander. For instance, a patient with CAR = 0.045 but a normal LAD may face comparable NOAF risk as another with a larger LAD but a low CAR. Recognizing such subclinical inflammatory sensitivity could improve risk stratification and promote earlier preventive strategies [4,26].

Our findings indicate that the prognostic threshold of LAD is context-dependent rather than universal in CCS. According to the American Society of Echocardiography (ASE) guidelines, normal anteroposterior LAD ranges from 30–40 mm in men and 27–38 mm in women, with values exceeding these considered abnormal [27]. Notably, current ASE/EACVI chamber quantification guidelines recommend left atrial volume, particularly when indexed to body surface area (LAVI), as the preferred parameter for atrial size assessment, because volumetric measures better capture asymmetric atrial remodeling and cumulative hemodynamic burden than a single anteroposterior diameter [28]. Prior studies have consistently shown that increased LA volume or LAVI is a stronger and more robust predictor of incident atrial fibrillation and adverse cardiovascular outcomes than linear LAD alone [29]. These findings suggest that mild increases in LAD may reflect early volumetric atrial remodeling that is not fully appreciated by linear measurements, providing a plausible explanation for the prognostic relevance of relatively modest LAD enlargement observed in the present CCS cohort. In our cohort, the exploratory LAD cutoff for predicting NOAF was 33.96 mm, lower than the conventional upper limit of 40 mm [30] but comparable to the 38.5 mm reported by Gerçek et al. for postoperative atrial fibrillation after CABG [31]. This convergence suggests that even mild atrial enlargement—sometimes within the guideline-defined “normal” range—may have prognostic relevance for arrhythmic risk in CCS. While this conclusion should be interpreted with moderate caution given the retrospective nature of the study, its consistency with prior findings reinforces the notion that structural vulnerability to AF may emerge at smaller atrial dimensions under conditions of chronic low-grade inflammation.

Sex, age, hypertension, diabetes mellitus, and hyperlipidemia are commonly used clinical strata when evaluating NOAF risk in patients with CCS. The effects of high CAR and enlarged LAD were most evident in older patients and those with hypertension, conditions linked to inflammation and atrial fibrosis. Extended analyses using combined CAR–LAD categories revealed similar trends but with wider confidence intervals due to smaller subgroup sizes (Table S1). Overall, these results indicate that inflammatory and structural factors provide complementary insights into atrial vulnerability in CCS. However, these subgroup analyses were exploratory in nature, and the findings should be interpreted cautiously and require independent validation in future studies.

The observed associations are consistent with the dual “trigger–substrate” hypothesis that underpins AF pathogenesis [11]. As initiating triggers, myocardial ischemia, oxidative stress, and systemic inflammation collectively contribute to atrial electrical instability, fibrosis, and autonomic imbalance [32]. Within this framework, several inflammation-based indices—such as the neutrophil-to-lymphocyte ratio (NLR) and the systemic immune-inflammation index (SII)— have been examined; however, their predictive performance has been inconsistent and generally limited [33,34,35]. Although these indices effectively capture systemic inflammatory activity, their predictive utility remains heterogeneous and frequently suboptimal. By contrast, CAR, derived from CRP and albumin, provides a more integrated representation of both inflammatory and nutritional status. Prior studies have suggested that it surpasses CRP alone in predicting cardiovascular risk [34,36,37].

Atrial enlargement serves as the structural substrate for the maintenance of AF [38]. LAD, reflecting the cumulative hemodynamic load, has long been associated with incident AF, heart failure, and mortality [37,38,39]. It should be recognized, however, that LA enlargement primarily reflects a relatively late manifestation of atrial remodeling, whereas earlier stages of atrial dysfunction may be better captured by functional parameters such as left atrial reservoir strain [40]. Most prior investigations linking inflammation and atrial size with NOAF have been conducted in ACS or perioperative populations, where inflammatory stress is markedly higher [41,42]. Our findings extend this evidence to CCS, showing that even under sustained, low-grade inflammation, the interaction between inflammatory activity and structural remodeling remains clinically relevant. Taken together, CAR and LAD capture the inflammatory “trigger” and structural “substrate” of AF, respectively. Although more advanced functional atrial indices were not available in the present cohort, the combined CAR–LAD model achieved moderate but clinically meaningful discrimination and offers practical value for risk stratification in CCS patients.

Beyond CAR and LAD, conventional cardiovascular risk factors remain central to AF pathogenesis. Importantly, these baseline clinical characteristics were not interpreted in isolation, as all relevant covariates were systematically adjusted for in the multivariable Cox regression models to mitigate potential confounding. In the CLARIFY registry, older age, higher BMI, impaired renal function, and reduced LVEF were all significantly associated with incident AF, with hazard ratios ranging from approximately 1.3 to 2.5 [4]. Furthermore, cardiometabolic comorbidities—including hypertension, diabetes, dyslipidemia, and smoking—have been shown to promote atrial remodeling and inflammation, predisposing CCS patients to AF [43]. Although these factors were not the focus of our analysis, they provide important context for interpreting our findings and suggest that combining inflammation- and structure-based markers with traditional risk factors may yield the most refined risk stratification in CCS.

From a clinical perspective, CAR and LAD are practical, low-cost biomarkers readily available from routine assessments. Their combined use may facilitate identification of CCS patients at higher risk of NOAF, supporting closer rhythm surveillance and optimized risk management. Decision curve analysis further demonstrated that integrating CAR and LAD with clinical factors provided the greatest net clinical benefit, highlighting their potential for individualized prediction. In this context, established tools such as the CHARGE-AF score—and other community-based AF risk models including the Framingham AF score—have been developed to predict incident atrial fibrillation primarily using demographic and clinical variables [44,45]. However, these models generally do not incorporate markers of systemic inflammation or direct measures of atrial structure. Our findings suggest that CAR and LAD capture complementary inflammatory and structural domains not explicitly represented in existing AF prediction tools and may, therefore, provide incremental value when integrated into such frameworks, particularly in CCS. Incorporating these parameters into future risk stratification frameworks could refine personalized management of CCS, although prospective validation will be essential before clinical implementation [46]. Notably, CAR and LAD serve a different purpose from traditional scores such as CHA_2_DS_2_-VASc and HATCH, which estimate thromboembolic or recurrence risk in established AF. Conversely, CAR and LAD focus on predicting AF onset, capturing upstream inflammatory and structural susceptibility. Prior studies have shown that adding LAD improves thromboembolic risk prediction [47] and that combining inflammatory markers with left atrial volume index enhances recurrence prediction [48]. Extending this paradigm, our findings suggest that combining CAR and LAD could refine individualized risk stratification in CCS.

This study delineates several research boundaries that also define future directions for investigation. First, as a retrospective single-center analysis, NOAF identification relied on discharge diagnoses and available examination records. This design reflects real-world clinical practice but underscores the need for prospective, continuous rhythm monitoring to detect transient or early post-discharge arrhythmia events. As a result, asymptomatic or short-duration AF episodes may have been missed, and potential misclassification bias in NOAF ascertainment cannot be excluded. Second, the present work focused on LAD as a representative structural parameter. The lack of data on atrial geometry, volume, and AF burden limited comprehensive assessment of atrial remodeling [49]. Moreover, left atrial size was assessed using absolute LAD rather than body size-indexed parameters (e.g., LAD/BSA or LAVi). Given potential fluctuations in body surface area related to fluid status or metabolic treatment in CCS patients, indexed measurements may introduce additional variability; nevertheless, future studies incorporating indexed, volumetric, and functional atrial parameters—such as left atrial reservoir strain—are warranted. Third, detailed longitudinal information on medication use—including statins, aspirin, ACEI/ARB, β-blockers, SGLT2 inhibitors, metformin, ezetimibe, and other agents known to influence systemic inflammation or CRP levels—was unavailable. CCS patients often have multiple comorbidities and dynamic treatment regimens, making it difficult to reliably capture medication initiation, discontinuation, dose adjustments, or adherence over time. Consequently, the modifying effects of CRP-related pharmacotherapy on NOAF risk could not be fully assessed, and residual confounding from anti-inflammatory or cardiometabolic medications cannot be excluded. Baseline lipid differences may largely reflect variations in statin intensity or other lipid-lowering therapies rather than inherent biological differences. Fourth, CRP is a dynamic inflammatory biomarker that may fluctuate over time due to intercurrent illness or medical therapy. In this study, CRP was measured at baseline only, and, therefore, the long-term prognostic value of a single CRP measurement should be interpreted with caution. Collectively, these limitations highlight opportunities to explore how inflammatory activity, structural remodeling, and metabolic regulation interact to drive arrhythmogenesis in CCS.

Looking ahead, future AF research in CCS should adopt a dynamic, integrative, and patient-centered prediction. Prospective multicenter studies combining longitudinal CAR trajectories with advanced atrial imaging may capture the evolving interplay between inflammation and structural remodeling. Future trials should also examine whether targeted interventions—anti-inflammatory therapy, renin–angiotensin–aldosterone system inhibition, or lifestyle modification—can truly alter this trajectory and prevent NOAF onset. At the intersection of systems immunology, metabolic science, and computational modeling, emerging technologies may enable individualized “digital twin” prediction models that translate biological mechanisms into real-time clinical guidance.

In summary, this study establishes a pragmatic link between systemic inflammation and atrial remodeling in CCS. The combined use of CAR and LAD—simple, reproducible, and widely available parameters—offers a practical means for early identification of patients at increased risk of NOAF. Beyond improving risk prediction, these findings highlight the potential of incorporating routine biomarkers into preventive cardiology, facilitating earlier intervention and tailored surveillance even in resource-limited settings. As cardiovascular care moves toward precision and prevention, translating such accessible indicators into clinical decision-making frameworks may help bridge the gap between population-level risk and individualized management, advancing timely and equitable strategies for AF prevention across the spectrum of coronary artery disease.

## 5. Conclusions

Among CCS patients, NOAF occurred in 3.80% during follow-up. Both CAR and LAD independently predicted NOAF, and their combination yielded superior discriminatory performance (AUC = 0.709). The occurrence of NOAF was associated with an increased cardiovascular risk, underscoring its clinical and economic burden. These findings support CAR–LAD integration as a simple and pragmatic tool for early risk stratification in CCS; however, external validation in independent cohorts is required before routine clinical adoption. Prospective multicenter studies are warranted to determine whether CAR–LAD-guided preventive strategies and systematic rhythm monitoring can reduce NOAF incidence and improve long-term cardiovascular outcomes.

## Figures and Tables

**Table 1:** Clinical characteristics of the study population stratified by NOAF.

Characteristic	Total Patients (N = 2431)	NOAF		*p*-Value
		No (N = 2338)	Yes (N = 93)	
Demographic characteristics				
Male, n (%)	1412 (58.1)	1353 (57.9)	59 (63.4)	0.351
Age, (y)	67.0 [60.0;74.0]	67.0 [60.0;74.0]	74.0 [69.0;77.0]	**<0.001**
Current drinker, n (%)	408 (16.8)	395(17.0)	13 (14.0)	0.544
SBP (mmHg)	140 [126;154]	140 [126;154]	143 [129;158]	0.227
DBP (mmHg)	78.0 [70.0;86.0]	78.0 [70.0;86.0]	74.0 [70.0;82.0]	**0.031**
History of coronary revascularization, n (%)				
PCI, n (%)	653 (26.9)	637 (27.2)	16 (17.2)	**0.032**
CABG, n (%)	9 (0.4)	9 (0.4)	0 (0.0)	1.000
None, n (%)	1769 (72.8)	1692 (72.4)	77 (82.8)	**0.027**
Comorbidities				
History of myocardial infarction, n (%)	173 (7.1)	166 (7.1)	7 (7.5)	0.875
Hypertension, n (%)	1659 (68.2)	1593 (68.1)	66 (71.0)	0.642
Hyperhomocysteinemia, n (%)	40 (1.65)	38 (1.63)	2 (2.15)	0.663
Diabetes mellitus, n (%)	1039 (42.7)	1000 (42.8)	39 (41.9)	0.982
Hyperlipidemia, n (%)	399 (16.4)	390 (16.7)	9 (9.68)	0.106
Hyperuricemia, n (%)	202 (8.29)	196 (8.38)	6 (6.45)	0.643
Laboratory parameters				
TC (mmol/L)	4.36 [3.59;5.26]	4.38 [3.60;5.29]	4.09 [3.38;4.72]	**0.003**
TG (mmol/L)	1.31 [0.93;1.85]	1.32 [0.93;1.87]	1.16 [0.89;1.53]	**0.012**
HDL-C (mmol/L)	1.04 [0.88;1.24]	1.04 [0.88;1.24]	1.06 [0.89;1.21]	0.94
LDL-C (mmol/L)	2.67 [2.01;3.40]	2.68 [2.02;3.41]	2.53 [1.82;3.00]	**0.022**
RLPC (mmol/L)	0.52 [0.34;0.77]	0.52 [0.34;0.78]	0.45 [0.32;0.61]	**0.025**
Lpa (mmol/L)	144 [75.0;288]	144 [76.0;288]	122 [64.7;290]	0.343
ApoB100 (mmol/L)	1.02 [0.79;1.29]	1.02 [0.79;1.30]	0.95 [0.69;1.17]	**0.003**
K^+^ (mmol/L)	3.95 [3.71;4.20]	3.95 [3.71;4.20]	3.90 [3.67;4.16]	0.38
WBC (×109/L)	6.80 [5.66;8.26]	6.80 [5.66;8.25]	6.85 [5.92;8.40]	0.763
NEU (×109/L)	4.10 [3.22;5.34]	4.09 [3.22;5.33]	4.22 [3.13;5.71]	0.461
CRP (mg/L)	4.33 [1.00;22.8]	4.20 [1.00;22.3]	10.4 [2.09;32.5]	**0.003**
FBG (mmol/L)	5.72 [4.99;7.17]	5.72 [4.98;7.16]	5.93 [5.16;7.21]	0.468
ALB (g/L)	40.1 [37.7;42.6]	40.2 [37.7;42.7]	39.4 [37.5;41.4]	**0.030**
BUN (mmol/L)	5.61 [4.58;7.04]	5.58 [4.57;6.99]	6.50 [5.14;8.16]	**<0.001**
Cr (μmol/L)	79.0 [66.0;95.0]	79.0 [65.9;95.0]	85.0 [72.0;106]	**0.003**
UA (μmol/L)	377 [307;456]	376 [306;455]	411 [331;481]	**0.049**
HbA1c (%)	6.20 [5.70;7.30]	6.20 [5.70;7.39]	6.10 [5.60;6.70]	0.15
TyG	7.17 [6.75;7.59]	7.17 [6.75;7.59]	7.05 [6.77;7.36]	0.077
SHR	0.81 [0.70;0.94]	0.81 [0.70;0.94]	0.82 [0.69;0.96]	0.593
AIP	0.10 [−0.08;0.29]	0.10 [−0.08;0.30]	0.06 [−0.09;0.22]	0.083
CAR	0.11 [0.02;0.60]	0.11 [0.02;0.59]	0.24 [0.05;0.91]	**0.004**
Coronary angiography characteristics				
Coronary angiography available, n (%)	2071 (85.2)	1993 (85.2)	78 (83.9)	0.715
Number of vessels with ≥50% stenosis, n (%)				
0-vessel disease	594 (28.7)	570 (28.6)	24 (30.8)	0.678
1-vessel disease	684 (33.0)	661 (33.2)	23 (29.5)	0.498
2-vessel disease	428 (20.7)	414 (20.8)	14 (17.9)	0.546
≥3-vessel disease	365 (17.6)	348 (17.5)	17 (21.8)	0.324
Multi-vessel disease (≥2 vessels), n (%)	793 (38.3)	762 (38.2)	31 (39.7)	0.788
Left main disease (≥50%), n (%)	127 (6.1)	123 (6.2)	4 (5.1)	1.000
Echocardiography results				
LVEF (%)	67.0 [62.7;71.0]	67.0 [63.0;71.0]	66.0 [60.0;70.0]	0.130
LAD (mm)	32.0 [30.0;36.0]	32.0 [30.0;36.0]	36.0 [33.0;40.0]	**<0.001**
Follow-up duration for NOAF (days)	1810 [1151;2321]	1846 [1205;2334]	832 [555;1280]	**<0.001**

Bold values indicate significant difference between two groups (*p* < 0.05). TC: Total cholesterol; TG: Triglycerides; HDL-C: High-density lipoprotein cholesterol; LDL-C: Low-density lipoprotein cholesterol; RLPC: Remnant-like particle cholesterol; Lpa: Lipoprotein(a); ApoB100: Apolipoprotein B100; K^+^: Serum potassium; WBC: White blood cell count; NEU: Neutrophil count; CRP: C-reactive protein; FBG: Fasting blood glucose; ALB: Albumin; BUN: Blood urea nitrogen; Cr: Creatinine; UA: Uric acid; HbA1c: Glycated hemoglobin; TyG: Triglyceride-glucose index; SHR: Stress hyperglycemia ratio; AIP: Atherogenic index of plasma; CAR: C-reactive protein to albumin ratio. NOAF: New-Onset Atrial Fibrillation. Continuous variables were described as mean ± standard deviation (SD) if normally distributed, or as median with interquartile range (IQR) if not normally distributed. Categorical variables were presented as numbers and percentages. Coronary angiography data were missing in 14.8% of patients. Percentages were calculated based on available data.

**Table 2:** The predictive value of predictor factor for NOAF in patients.

Variables	ROC-Derived Cutoff	Sensitivity (%)	Specificity (%)	AUC (95%CI)	*p* Value
CAR	0.043	0.352	0.796	0.587 (0.532–0.644)	**0.002**
LAD	33.96	0.657	0.656	0.692 (0.640–0.754)	**<0.001**
CAR and LAD	0.004	0.601	0.731	0.709 (0.654–0.765)	**<0.001**

Bold values indicate significant difference between two groups (*p* < 0.05). LAD: Left atrial diameter; CAR: C-reactive Protein to Albumin Ratio. ROC-derived cutoff is determined by ROC curve analysis using the maximal Youden index.

**Table 3:** Comparison of baseline characteristics by CAR index and LAD grouping.

Characteristic	Total Patients (N = 2431)	Group 1 (N= 581)	Group 2 (N = 982)	Group 3 (N = 267)	Group 4 (N = 601)	*p*-Value
Demographic characteristics						
Male, n (%)	1412 (58.1)	344 (59.2%)	563 (57.3%)	166 (62.2%)	339 (56.4%)	0.412
Age, (y)	67.0 [60.0;74.0]	65.0 [58.0;72.0]	67.0 [60.0;74.0]	68.0 [59.0;75.0]	69.0 [62.0;75.0]	**<0.001**
Current drinker, n (%)	408 (16.8)	104 (17.9%)	163 (16.7%)	50 (18.8%)	91 (15.3%)	0.494
SBP (mmHg)	140 [126;154]	137 [125;150]	139 [126;154]	141 [127;153]	142 [128;158]	**0.001**
DBP (mmHg)	78.0 [70.0;86.0]	79.0 [70.0;86.0]	78.0 [71.0;87.0]	77.0 [69.0;84.0]	77.0 [69.0;86.0]	0.059
Comorbidities						
Hypertension, n (%)	1659 (68.2)	362 (62.3%)	676 (68.8%)	186 (69.7%)	435 (72.4%)	**0.002**
Hyperhomocysteinemia, n (%)	40 (1.65)	11 (1.89%)	13 (1.30%)	8 (3.00%)	8 (1.33%)	0.228
Diabetes mellitus, n (%)	1039 (42.7)	245 (42.2%)	395 (40.2%)	112 (41.9%)	287 (47.8%)	**0.022**
Hyperlipidemia, n (%)	399 (16.4)	104 (17.9%)	186 (18.9%)	42 (15.7%)	67 (11.1%)	**0.001**
Hyperuricemia, n (%)	202 (8.29)	48 (8.26%)	86(8.76%)	22 (8.24%)	46 (7.65%)	0.909
Laboratory parameters						
TC (mmol/L)	4.36 [3.59;5.26]	4.42 [3.69;5.27]	4.44 [3.58;5.36]	4.14 [3.50;4.98]	4.30 [3.56;5.26]	**0.016**
TG (mmol/L)	1.31 [0.93;1.85]	1.31 [0.92;1.74]	1.31 [0.93;1.89]	1.29 [0.93;1.75]	1.32 [0.92;1.92]	0.531
HDL-C (mmol/L)	1.04 [0.88;1.24]	1.09 [0.92;1.28]	1.04 [0.88;1.23]	1.02 [0.87;1.24]	0.99 [0.86;1.18]	**<0.001**
LDL-C (mmol/L)	2.67 [2.01;3.40]	2.67 [2.05;3.40]	2.72 [2.02;3.44]	2.48 [1.88;3.17]	2.64 [2.01;3.42]	**0.022**
RLPC (mmol/L)	0.52 [0.34;0.77]	0.49 [0.32;0.72]	0.53 [0.34;0.80]	0.52 [0.32;0.75]	0.53 [0.34;0.78]	0.074
Lpa (mmol/L)	144 [75.0;288]	127 [71.0;261]	144 [80.0;292]	127 [63.0;267]	169 [80.0;306]	**0.005**
ApoB100 (mmol/L)	1.02 [0.79;1.29]	1.02 [0.78;1.27]	1.04 [0.81;1.32]	0.97 [0.74;1.23]	1.03 [0.79;1.29]	**0.024**
K^+^ (mmol/L)	3.95 [3.71;4.20]	3.95 [3.72;4.18]	3.95 [3.70;4.19]	3.97 [3.70;4.24]	3.95 [3.71;4.23]	0.779
WBC (×109/L)	6.80 [5.66;8.26]	6.39 [5.37;7.65]	7.01 [5.74;8.51]	6.59 [5.59;7.92]	6.95 [5.82;8.65]	**<0.001**
NEU (×109/L)	4.10 [3.22;5.34]	3.70 [2.93;4.65]	4.27 [3.32;5.60]	3.89 [3.21;4.98]	4.36 [3.46;5.61]	**<0.001**
CRP (mg/L)	4.33 [1.00;22.8]	0.60 [0.20;1.10]	14.7 [4.60;35.7]	0.70 [0.25;1.18]	14.3 [4.40;35.6]	**<0.001**
FBG (mmol/L)	5.72 [4.99;7.17]	5.52 [4.97;6.77]	5.74 [4.98;7.20]	5.67 [4.96;7.22]	5.99 [5.06;7.46]	**0.001**
ALB (g/L)	40.1 [37.7;42.6]	41.2 [38.9;43.1]	40.0 [37.6;42.5]	40.8 [38.5;43.0]	39.1 [36.3;41.2]	**<0.001**
BUN (mmol/L)	5.61 [4.58;7.04]	5.37 [4.43;6.51]	5.48 [4.53;6.75]	5.85 [4.76;7.18]	6.06 [4.86;7.97]	**<0.001**
Cr (μmol/L)	79.0 [66.0;95.0]	75.0 [63.0;90.0]	78.0 [65.0;94.0]	80.0 [68.7;94.5]	84.0 [68.0;107]	**<0.001**
UA (μmol/L)	377 [307;456]	363 [304;433]	368 [298;449]	399 [328;469]	398 [322;486]	**<0.001**
HbA1c (%)	6.20 [5.70;7.30]	6.10 [5.60;7.28]	6.20 [5.60;7.30]	6.10 [5.70;6.90]	6.40 [5.80;7.60]	**<0.001**
TyG	7.17 [6.75;7.59]	7.11 [6.71;7.50]	7.19 [6.75;7.63]	7.13 [6.74;7.55]	7.21 [6.79;7.63]	**0.046**
SHR	0.81 [0.70;0.94]	0.79 [0.70;0.90]	0.82 [0.71;0.97]	0.81 [0.70;0.94]	0.79 [0.69;0.95]	**0.037**
AIP	0.10 [−0.08;0.29]	0.07 [−0.11;0.23]	0.12 [−0.08;0.30]	0.08 [−0.10;0.28]	0.12 [−0.06;0.30]	**0.003**
LVEF (%)	67.0 [62.7;71.0]	68.0 [64.0;72.0]	67.0 [63.0;71.0]	66.0 [60.0;70.0]	65.0 [60.0;70.0]	**<0.001**
Outcome						
NOAF						**<0.001**
No	2338 (96.2%)	574 (98.8%)	975 (97.4%)	255 (95.5%)	552 (91.8%)	
Yes	93 (3.8%)	7 (1.20%)	25 (2.50%)	12 (4.49%)	49 (8.15%)	

Bold values indicate significant difference between two groups (*p* < 0.05). TC: Total cholesterol; TG: Triglycerides; HDL-C: High-density lipoprotein cholesterol; LDL-C: Low-density lipoprotein cholesterol; RLPC: Remnant-like particle cholesterol; Lpa: Lipoprotein(a); ApoB100: Apolipoprotein B100; K^+^: Serum potassium; WBC: White blood cell count; NEU: Neutrophil count; CRP: C-reactive protein; FBG: Fasting blood glucose; ALB: Albumin; BUN: Blood urea nitrogen; Cr: Creatinine; UA: Uric acid; HbA1c: Glycated hemoglobin; TyG: Triglyceride-glucose index; SHR: Stress hyperglycemia ratio; AIP: Atherogenic index of plasma; CAR: C-reactive protein to albumin ratio. NOAF: New-Onset Atrial Fibrillation. Continuous variables were described as mean ± standard deviation (SD) if normally distributed, or as median with interquartile range (IQR) if not normally distributed. Categorical variables were presented as numbers and percentages. Patients were categorized into four groups according to the ROC-derived cutoffs of CAR (0.0429) and LAD (33.96 mm): Group 1, low CAR (<0.0429) and low LAD (<33.96 mm); Group 2, high CAR (≥0.0429) and low LAD (<33.96 mm); Group 3, low CAR (<0.0429) and high LAD (≥33.96 mm); and Group 4, high CAR (≥0.0429) and high LAD (≥33.96 mm).

**Table 4:** COX regression models for the association of the CAR index and LAD with the incidence of NOAF in CCS patients.

Characteristics	Model 1		Model 2		Model 3	
	HR (95% CI)	*p* Value	HR (95% CI)	*p* Value	HR (95% CI)	*p* Value
**CAR**						
Continuous (per SD)	1.21 (1.02–1.44)	**0.025**	1.14 (0.95–1.35)	0.147	1.12 (0.95–1.34)	0.177
CAR < 0.0429, n = 848	ref		ref		ref	
CAR ≥ 0.0429, n = 1583	2.02 (1.22–3.36)	**0.006**	1.80 (1.09–2.99)	**0.021**	1.85 (1.11–3.08)	**0.018**
**LAD**						
Continuous (per SD)	1.13 (1.10–1.17)	**<0.001**	1.12 (1.09–1.16)	**<0.001**	1.13 (1.08–1.17)	**<0.001**
LAD < 33.96 mm, n = 1563	ref		ref		ref	
LAD ≥ 33.96 mm, n = 868	3.39 (2.21–5.20)	**<0.001**	3.04 (1.98–4.67)	**<0.001**	2.87 (1.84–4.48)	**<0.001**
**CAR and LAD**						
Continuous (per SD)	2.71 (2.12–3.49)	**<0.001**	2.58 (1.99–3.37)	**<0.001**	2.67 (1.99–3.57)	**<0.001**
Group 1 (Low CAR and low LAD; n = 581)	ref		ref		ref	
Group 2 (high CAR and Low LAD; n = 982)	2.04 (0.88–4.72)	0.094	1.75 (0.76–4.06)	0.189	1.74 (0.75–4.05)	0.195
Group 3 (low CAR and High LAD; n = 267)	3.65 (1.44–9.28)	**0.006**	3.01 (1.18–7.68)	**0.021**	2.69 (1.05–6.91)	**0.040**
Group 4 (High CAR and high LAD; n = 601)	6.52 (2.95–14.39)	**<0.001**	5.25 (2.37–11.61)	**<0.001**	5.05 (2.26–11.31)	**<0.001**
*p* for trend	**<0.001**		**<0.001**		**<0.001**	

Model 1: Unadjusted. Model 2: Adjusted for age and sex. Model 3: Adjusted for age, sex, systolic blood pressure, apolipoprotein B100, creatinine, potassium, uric acid, total cholesterol, glycated hemoglobin, drinking history, diabetes mellitus history, pulse, left ventricular ejection fraction (LVEF), stroke history, and hyperhomocysteinemia history. CAR = C-reactive protein–to–albumin ratio; LAD = left atrial diameter. Bold values indicate significant difference between two groups (*p* < 0.05). HR indicates Hazard Ratios; SD indicates standard deviation.

## Data Availability

The data supporting the findings of this study can be obtained from the corresponding author upon a reasonable request. The data is inaccessible to the public owing to privacy or ethical constraints.

## Supplementary Materials

The following supporting information can be downloaded at: https://www.mdpi.com/article/10.3390/jcm15010255/s1, Table S1: Subgroup analyses of the association between combined CAR–LAD grouping and new-onset atrial fibrillation; Table S2. Definitions of Study Variables; Method S1. Validation of ICD Codes for AF/AFL; Method S2. Handling of Missing Data. STROBE Statement—Checklist of items that should be included in reports of cohort studies.
