# Supplementary material for: C-Reactive Protein-to-Albumin Ratio (CAR) and Left Atrial Diameter Predicts New-Onset Atrial Fibrillation in Chronic Coronary Syndrome: A Retrospective Cohort Study
Source: J Clin Med. 2025 Dec 29;15(1):255. doi: 10.3390/jcm15010255 (PMC12787201; doi:10.3390/jcm15010255)
Supplement: Supplementary file 1 [file jcm-15-00255-s001.zip › jcm-4030433 Supplementary Materials Table.pdf]

Table S1 Subgroup analyses of the association between combined CAR－LAD grouping and new-onset atrial fibrillation.

Stratified variable	Group 1	*P* value	Group 2	*P* value	Group 3	*P* value	Group 4	*P* value	*P* for interaction
Sex									0.321
Male	Ref.	-	1.27 (0.50-3.25)	0.620	2.26 (0.79-6.46)	0.129	3.38 (1.37-8.33)	**0.008**	
Female	Ref.	-	4.80 (0.59-38.88)	0.141	5.49 (0.56-54.25)	0.144	1.79 (2.33-137.87)	**0.005**	
Age									0.654
<65	Ref.	-	0.79 (0.11-5.80)	0.813	1.08 (0.09-12.47)	0.949	5.80 (1.06-31.69)	**0.042**	
≥65	Ref.	-	2.09 (0.79-5.53)	0.137	3.32 (1.14-9.69)	**0.027**	5.57 (2.18-14.23)	**<0.001**	
Diabetes mellitus									0.815
No	Ref.	-	2.06 (0.68-6.17)	0.196	2.61 (0.72-9.43)	0.142	5.85 (2.00-17.14)	**0.001**	
Yes	Ref.	-	1.51 (0.39-5.77)	0.547	2.97 (0.71-12.32)	0.134	4.04 (1.20-13.64)	**0.024**	
Hypertension									0.660
No	Ref.	-	4.85 (0.60-39.13)	0.137	7.63 (0.83-70.40)	0.073	12.44 (1.56-98.67)	**0.017**	
Yes	Ref.	-	1.31 (0.51-3.34)	0.573	1.99 (0.68-5.82)	0.206	3.84 (1.60-9.24)	**0.002**	
Hyperlipidemia									0.513
No	Ref.	-	2.13 (0.80-5.64)	0.130	3.08 (1.04-9.13)	**0.043**	6.27 (2.45-16.02)	**<0.001**	
Yes	Ref.	-	1.16 (0.17-7.84)	0.878	1.04 (0.08-13.69)	0.974	3.64 (0.30-43.99)	0.308	

Subgroup analyses based on sex, age, diabetes mellitus (DM), hypertension, and hyperlipidemia were conducted to evaluate the incidence of NOAF across four combined categories of baseline C-reactive protein-to-albumin ratio (CAR) and left atrial diameter (LAD): Group 1 (low CAR–low LAD), Group 2 (high CAR–low LAD), Group 3 (low CAR–high LAD), and Group 4 (high CAR–high LAD). *P* for interaction indicates the significance of the interaction term between CAR–LAD grouping and each stratified variable.

Bold values indicate statistical significance ($P < 0.05$).

CI = confidence interval; HR = hazard ratios; CAR = C-reactive protein-to-albumin ratio; LAD = left atrial diameter.

Table S2. Definitions of Study Variables

Variable	Definition	Unit / Category	Timing
CCS	Chronic coronary syndrome defined according to 2019 ESC guidelines	—	Baseline
NOAF	First documented occurrence of AF or AFL confirmed by ECG or medical records	Yes / No	During follow-up
AF	Atrial fibrillation documented by ECG or physician records	Yes / No	During follow-up
AFL	Atrial flutter documented by ECG or physician records	Yes / No	During follow-up
CAR	C-reactive protein (mg/L) divided by serum albumin (g/L)	Ratio	Baseline
LAD	Left atrial diameter measured by transthoracic echocardiography (parasternal long-axis view)	mm	Baseline
RLPC	Remnant lipoprotein cholesterol calculated as TC − LDL-C − HDL-C	mmol/L	Baseline
MI history	Prior myocardial infarction occurring >3 months before enrollment	Yes / No	Baseline
Revascularization	History of PCI or CABG performed >3 months before enrollment	Yes / No	Baseline / Follow-up
SBP	Systolic blood pressure measured at admission	mmHg	Baseline
LVEF	Left ventricular ejection fraction assessed by echocardiography	%	Baseline
Diabetes mellitus	Physician-diagnosed diabetes or use of antidiabetic medication	Yes / No	Baseline
Hyperhomocysteinemia	Serum homocysteine level above institutional reference range	Yes / No	Baseline

Method S1. Validation of ICD Codes for AF/AFL

Identification of new-onset atrial fibrillation (NOAF) and atrial flutter (AFL) was based on a multi-step validation strategy combining administrative codes and clinical record review. Potential AF/AFL cases were first identified using International Classification of Diseases (ICD) discharge diagnosis codes, including ICD-10 codes I48.0, I48.1, I48.2, I48.3, I48.4, and I48.9. Corresponding ICD-9 codes were also screened during the earlier study period. Patients with any prior AF or AFL diagnosis within the preceding 8 years were excluded to ensure identification of incident events only.

To ensure diagnostic accuracy, all AF/AFL cases identified through administrative codes were systematically validated by manual review of electronic medical records. Validation materials included inpatient 12-lead electrocardiograms, Holter monitoring or continuous cardiac rhythm monitoring records when available, and physician progress notes documenting AF or AFL episodes. A NOAF event was confirmed when at least one AF or AFL episode was documented by electrocardiographic evidence or clearly described in physician records. This multi-source validation approach has been routinely applied in our institution and is expected to yield a high positive predictive value for NOAF ascertainment.

Method S2. Handling of Missing Data

Missing data were evaluated for all baseline variables prior to statistical analysis. Overall, the proportion of missing data was low for most variables (0.24% for continuous variables and 1.24% for categorical variables). Coronary angiography data were missing in 14.8% of patients, primarily due to coronary angiography being performed at outside institutions, patient refusal of invasive procedures, or incomplete historical records.

Given the low overall proportion of missing data for key covariates, single imputation was applied to preserve the full study population for primary analyses. Continuous variables were imputed using the mean value, and categorical variables were imputed using the mode. The same imputation strategy was applied consistently across all baseline variables prior to further analyses. No imputation was performed for outcome variables.
